# Decadal surface temperature trends in India based on a new high-resolution data set

**DOI:** 10.1038/s41598-018-25347-2

**Published:** 2018-05-10

**Authors:** Robert S. Ross, T. N. Krishnamurti, Sandeep Pattnaik, D. S. Pai

**Affiliations:** 1 Department of Earth, Ocean and Atmospheric Science, Florida State University, Tallahassee, FL 32306 USA; 2 School of Earth, Ocean and Climate Sciences, Indian Institute of Technology, Bhubaneswar, Odisha 751013 India; 3 Indian Meteorological Department, Pune, Maharashtra 411005 India

## Abstract

A new comprehensive surface temperature data set for India is used to document changes in Indian temperature over seven decades, in order to examine the patterns and possible effects of global warming. The data set is subdivided into pre-monsoon, monsoon, and post-monsoon categories in order to study the temperature patterns in each of these periods. When the decade means in maximum, minimum and daily mean temperature for the 2000s are compared to those of the 1950s, a consistent pattern of warming is found over northwestern and southern India, and a pattern of cooling is seen in a broad zone anchored over northeastern India and extending southwestward across central India. These patterns are explained by the presence of a large region of anthropogenic brown haze over India and adjacent ocean regions. These aerosols absorb solar radiation, leading to warming of the haze layer over northeastern and central India and to cooling of the surface air beneath. The heated air rises and then sinks to the north and south of the haze region over northwestern and southern India, warming the air by compression as it sinks in those regions. The possible impact of these temperature patterns on Indian agriculture is considered.

## Introduction

The motivation for this study came from examination of all-India surface mean temperature anomalies for the period 1901–2016 shown in the supplementary information section of this paper as Fig. S1. These reveal an unmistakable rapid rise in Indian surface temperatures, particularly since about 1980, as seen in annual, winter, pre-monsoon, monsoon, and post-monsoon period depictions.

There has been great interest in India in recent decades concerning extreme values of temperature that have been observed, particularly during the warmest part of the year in April and May, the period preceding the onset of the summer monsoon. Such observations are consistent with global trends in temperature. The Intergovernmental Panel on Climate Change (IPCC) in its fifth assessment report^1^ reported that warming of the global climate system is unequivocal and this warming has accelerated since the 1950s. Each of the last three decades has been successively warmer at the earth’s surface than any prior decade based on records extending back to 1850. Globally averaged temperature for the land and ocean regions combined has shown an increase of 0.85 C since 1880.

Recent research^2^ has pointed out how climate change poses many challenges to growth and development in South Asia. India, for example, is more vulnerable to climate change because its agricultural system must feed 17.5% of the world’s population with only 2.4% of the land and 4% of the water resources of the planet. A mid-range projection of climate change for the period 2020–2039 indicates a crop yield reduction of 4.5–9% depending on the magnitude and distribution of the warming. Clearly it is extremely important to understand the patterns of long-term temperature change across India so that informed decisions can be made with respect to the demands on agricultural production.

In the current study, a new comprehensive temperature data set, unprecedented in both the number of observation stations involved and in its high horizontal resolution, has been used to document changes in Indian surface temperature over nearly seven decades, in order to examine the patterns and possible effects of global warming. This important new information on temperature trends across India has the potential to make significant contributions to future planning in the country, particularly for the agricultural system.

## Data and Methods

This research utilizes a recently developed comprehensive surface temperature data set for India for the period 1969–2005^3^. This data set has subsequently been expanded to include the period 1951–2013. The data set includes daily gridded maximum, minimum, and mean temperatures based on data from 395 Indian stations, interpolated onto a 1-degree latitude by 1-degree longitude grid.

In this study, the data set has been divided into three categories: the period from April 1 to May 31 which is the warmest part of the year (sometimes referred to as summer) occurring prior to the onset of the Indian summer monsoon; the period from July 1 to September 30 which represents the peak of the Indian monsoon; the period from January 1 to March 31 which is the coldest part of the year, coinciding with winter, and occurring well after the cessation of the Indian summer monsoon. Simple arithmetic means are constructed for each of these categories of the data set, for maximum, minimum, and daily mean temperature.

## Results: Patterns of Decadal Temperature Change

**Figure 1:**
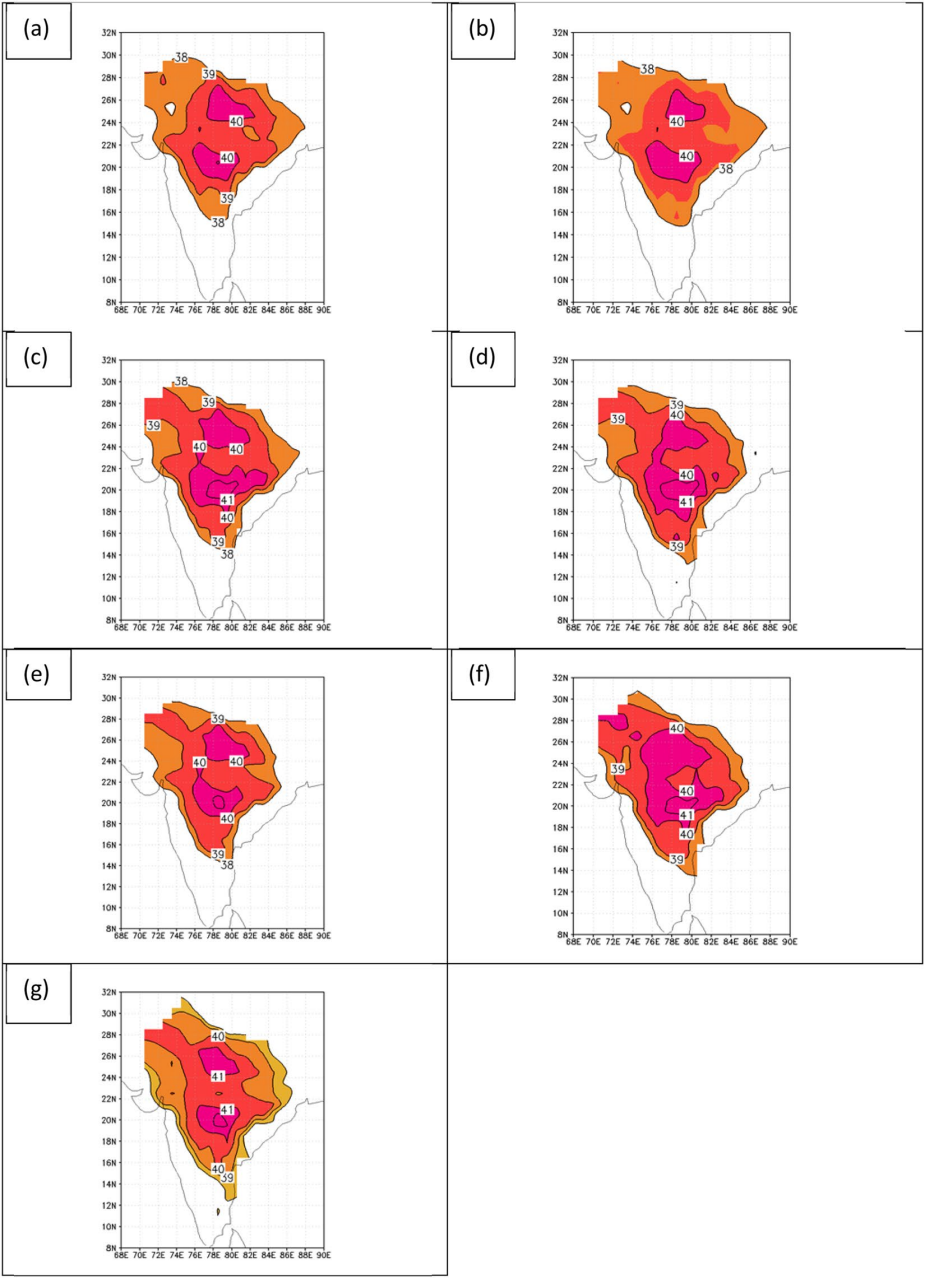
Decadal mean daily maximum temperature for the period April 1 to May 31. The decades shown are for (**a**) 1950s, (**b**) 1960s, (**c**) 1970s, (**d**) 1980s, (**e**) 1990s, (**f**) 2000s, and (**g**) 2010s. Figures are generated using GrADS 2.2.0 (http://cola.gmu.edu/grads/).

Decadal means of daily maximum temperature for the period April 1 to May 31 have been documented in this study to reach extreme values of 40 C (104 F) to 42 C (108 F) over parts of India. Figure 1(a–g) shows the maximum temperature means for this period for each decade from the 1950’s to the 2010’s. Contours are drawn for 38 C and greater, at a 1 C interval, to emphasize the increase in the peak values over time. In the 1950’s (panel a) the areas with mean maximum temperature greater than 40 C are limited with only a small spot in south-central India showing values as high as 41 C. The region with values greater than 40 C begins to expand in the 1970s and 1980s (panels c and d) and the region with values greater than 41 C in south-central India enlarges. After a very slight reduction in temperature in the 1990’s (panel e) the region with temperatures greater than 40 C expands dramatically in the 2000’s and the 2010’s (panels f and g), the region with temperatures greater than 41 C expands, and a region with temperatures greater than 42 C appears in south-central India in the 2010s (panel g). There is a notable warming trend in far northwestern India beginning in the 1970s (panel c) and accelerating in the 2000s and 2010s (panels f and g). These decadal mean temperature patterns suggest that global warming is manifesting itself over parts of India in the maximum temperatures observed during the warm pre-monsoon period, with an accelerating pace noted, particularly in the last two decades.

**Figure 2:**
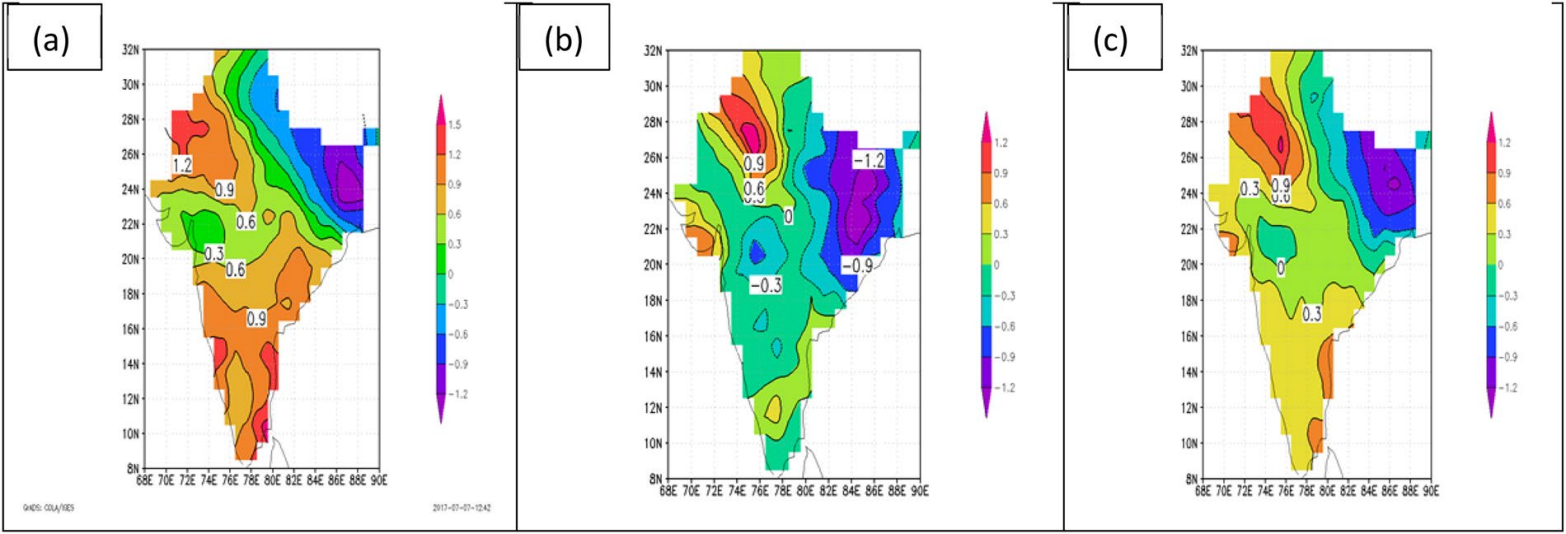
Difference in decade means of daily temperature (C) for the pre-monsoon period of April 1 to May 31. Values are for the decade of the 2000s minus the decade of the 1950s for (**a**) maximum temperature, (**b**) minimum temperature, and (**c**) mean temperature. Figures are generated using GrADS 2.2.0 (http://cola.gmu.edu/grads/).

Continuing to focus for the moment on the warm pre-monsoon period of April and May, three important patterns have been identified in the temperature data set when the decade of the 2000s is compared to the decade of the 1950s (2000s minus 1950s): (1) Warming is noted in northwest and southern India, with the greatest warming in the northwest; (2) A definite, and somewhat surprising, cooling (or in some instances, reduced warming) pattern is noted, which is anchored in northeastern India and which stretches southwestward across central India; (3) These patterns are generally seen in the maximum temperatures as well as the minimum temperatures, and, therefore, in the mean temperatures. These patterns are well illustrated in Fig. 2(a–c) which shows the difference in the decadal mean temperatures (daily maximum, daily minimum, daily mean, respectively) for the decade of the 2000’s minus the decade of the 1950’s. Note that in forming these temperature difference maps, all temperature values are used, not just values of 38 C and greater as in Fig. 1. This brings a wider geographical area into view as compared to Fig. 1. For maximum temperatures (panel a) the amplitude ranges from +1.5 C to −1.2 C, with minus values (cooling from 1950’s to 2000’s) confined to northeastern India, but with a swath of reduced warming extending southwestward across the country from this cooling region. The remainder of the country shows warming, with maxima in the northwest and southern regions. For minimum temperatures (panel b) the amplitude ranges from +1.2 C to −1.2 C, with minus values, greatest in northeastern India, extending across most of India, except for the far northwest region and the extreme southern region, where warming occurs, although this warming is less pronounced than that seen in the maximum temperatures (panel a). For daily mean temperatures (panel c) the amplitude range is comparable to that for the maximum and minimum temperatures, with warming in the northwest and southern regions, and cooling (or reduced warming) extending from the northeast region of India westward across the central part of the country. This pattern is consistent with the maximum and minimum temperature depictions.

**Figure 3:**
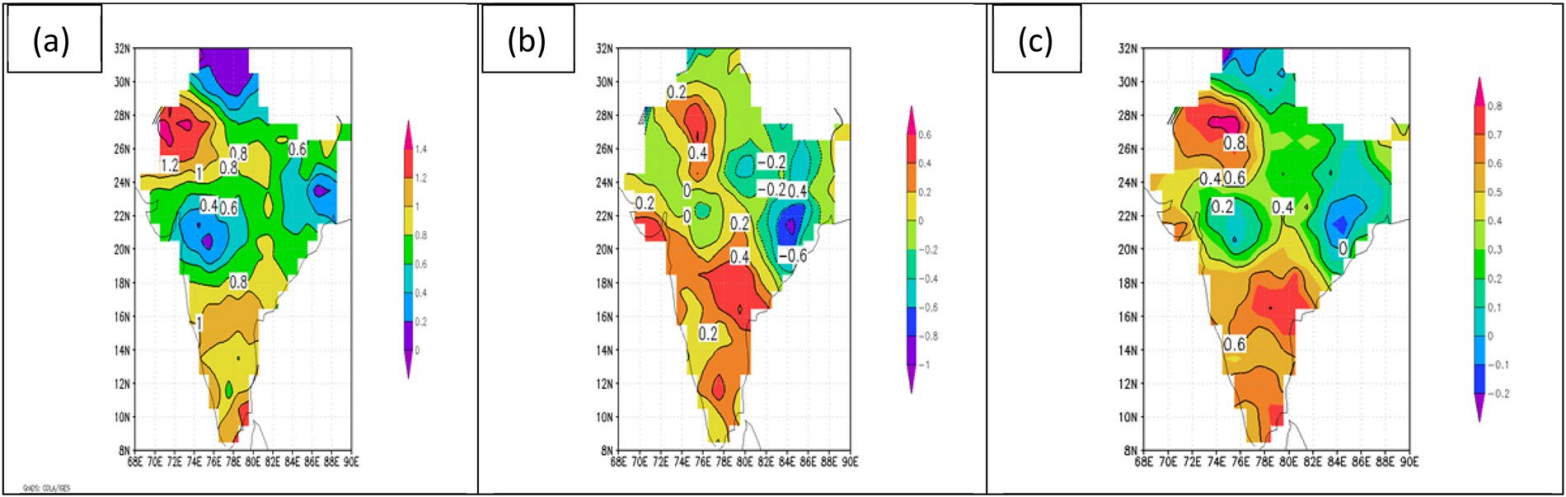
Difference in decade means of daily temperature (C) for the peak of the monsoon period of July 1 to September 30. Values are for the decade of the 2000s minus the decade of the 1950s for (**a**) maximum temperature, (**b**) minimum temperature, and (**c**) mean temperature. Figures are generated using GrADS 2.2.0 (http://cola.gmu.edu/grads/).

Turning to temperature patterns observed for the period of the peak of the monsoon (July 1 to September 30) the results are not qualitatively different from those for the pre-monsoon period. These results are shown in Fig. 3(a–c) for maximum temperature (panel a), minimum temperature (panel b), and daily mean temperature (panel c). These results are again for the decadal mean of the 2000’s minus the decadal mean for the 1950’s. One difference between Figs 2a and 3a for maximum temperatures is that the decadal temperature differences in the latter are positive everywhere except in the far northern portion of India, with no region of negative values in northeastern India as seen in Fig. 2a. However, there is region of reduced positive values extending from northeastern India southwestward across the country in Fig. 3a coinciding with the region of negative values (or reduced positive values) seen in Fig. 2a. For minimum temperatures during the peak of the monsoon (Fig. 3b), positive values (warming) are more extensive, particularly in southern India, as compared to Fig. 2b. Comparing daily mean temperatures in Fig. 2c versus Fig. 3c, the patterns are very similar but the amplitudes of the differences between the two decades are greater in Fig. 2c (pre-monsoon) than in Fig. 3c (peak of monsoon), with the amplitude ranging from −1.2 C to +1.2 C in the former and −0.2 C to +0.8 C in the latter.

**Figure 4:**
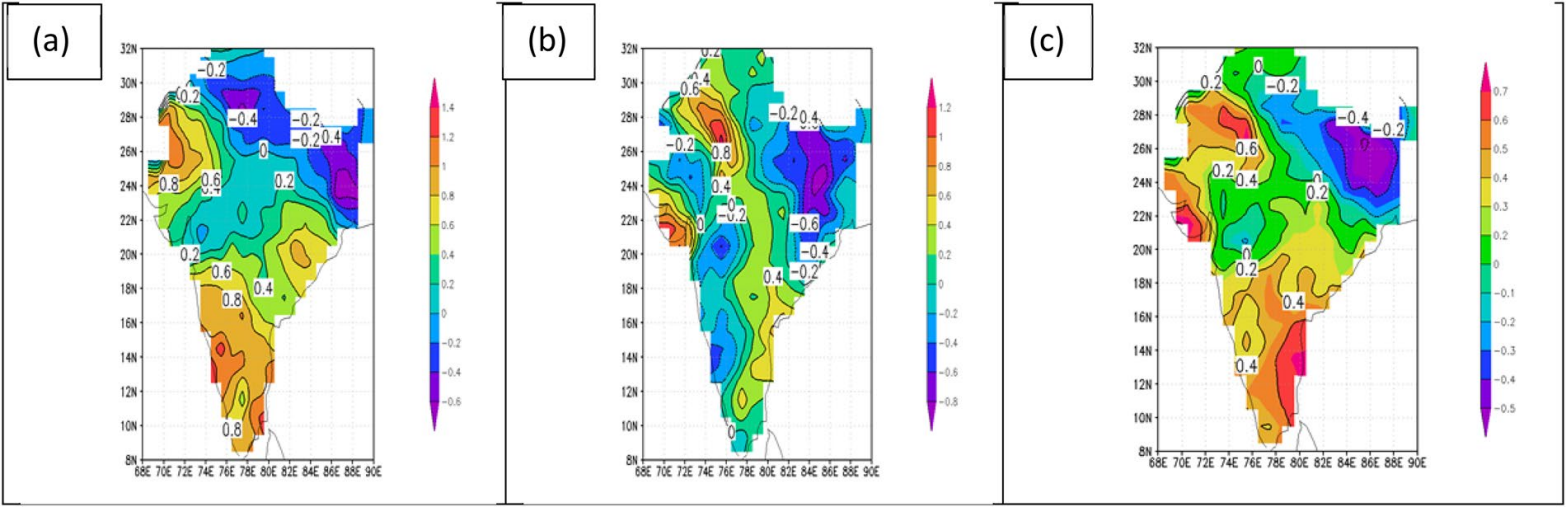
Difference in decade means of daily temperature (C) for the post-monsoon period of January 1 to March 31. Values are for the decade of the 2000s minus the decade of the 1950s for (**a**) maximum temperature, (**b**) minimum temperature, and (**c**) mean temperature. Figures are generated using GrADS 2.2.0 (http://cola.gmu.edu/grads/).

Considering temperature patterns observed during the coldest part of the year in India (January through March), the patterns are qualitatively the same as for the pre-monsoon and peak of the monsoon periods, but the amplitudes differ. The results for this coldest part of the year are shown in Fig. 4a−c, and as before represent the decadal means for the 2000s minus the decadal means for the 1950s. For maximum temperatures in winter (Fig. 4a) the pattern matches more closely the pattern for the pre-monsoon period (Fig. 2a) than the pattern for the height of the monsoon (Fig. 3a) due to the region of negative values dominating northeastern India in both maps. Otherwise, all three maps of maximum temperature are similar, showing positive values (warming) in northwest and southern India and reduced positive values (less warming) across central India. For minimum temperatures, the patterns in winter (Fig. 4b) once again match those of the pre-monsoon period (Fig. 2b) more closely than those of the peak of the monsoon (Fig. 3b). But the winter pattern shows a greater region of cooling, particularly over western India. In the maps of daily mean temperature for the decades of the 2000s minus the 1950s the patterns are remarkably similar for all three periods (comparing Figs 2c, 3c and 4c). These show warming regions over northwestern and southern India and a cooling pattern anchored over northeastern India, extending southwestward across central India.

**Figure 5:**
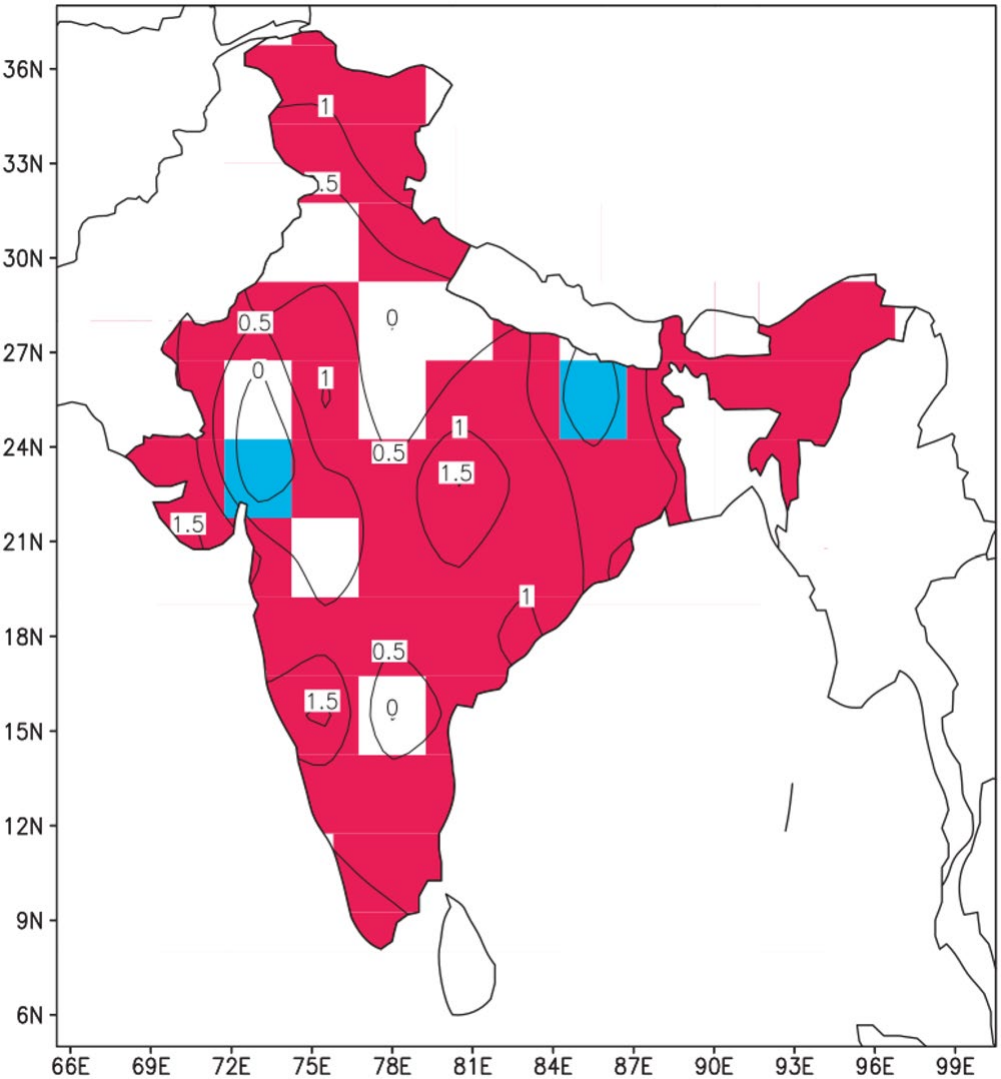
Annual mean temperature trends (C/100 years) over India for the period 1901–2016. Values are shown as contour lines. Trends significant at the 95% level are shaded, with positive trends shaded in red and negative trends shaded in blue. India Meteorological Department, Annual Climate Summary 2016, Pune, India.

Even though the temperature trend characterizations in Figs 2–4 are all based on the end-point decadal variations of the 2000s and the 1950s, nearly identical patterns were noted for comparisons of the decades of the 2010s to the 1950s, the 2000s to the 1960s, and the 1990s to the 1950s. These consistent temperature trend patterns are further confirmed by the spatial distribution of annual mean temperature trends for the period 1901–2016, as published by the Indian Meteorological Department and shown here as Fig. 5. The linear trend seen in this figure depicts significant warming over most of India but there are two regions of significant cooling, one to the east and one to the west in the region 23N to 26N. These patterns generally agree with those seen in Figs 2–4 for both warming and cooling regions.

From examination of all of these maps, the most salient question emerging is: What is causing the rather consistent patterns of warming and cooling over India as observed when comparing, for example, the decade of the 2000s to the decade of the 1950s, a 60-year span? This pattern consists of two warming regions over India, one to the northwest and one to the south, and a broad cooling region (or region of reduced warming), with greatest magnitude in northeastern India but with an extension southwestward into central India. This pattern is seen in all maps in Figs 2–4, except for maximum temperatures in Fig. 2a for the pre-monsoon period and in Fig. 3a during the peak of the monsoon, where there is no region of negative values (cooling) extending southwestward across India, but this region still shows reduced positive values (reduced warming). The question could also be stated as follows: With it well documented by countless studies that global warming is taking place on the planet, why is the warming pattern over India interrupted by the broad cooling pattern (or at least reduced warming pattern) that we see extending from northeast India southwestward across the country?

## Comparison to Other Studies

It is important to compare these results to other studies of long term changes in Indian surface temperatures. The present study reveals very similar temperature trends (symmetry) for both maximum and minimum temperatures. Diurnal asymmetries in temperatures have been reported for the period 1901–2003^4^. However, less diurnal asymmetry for the more recent period of 1971–2003 has been shown^5^, more in agreement with the present study.

**Figure 6:**
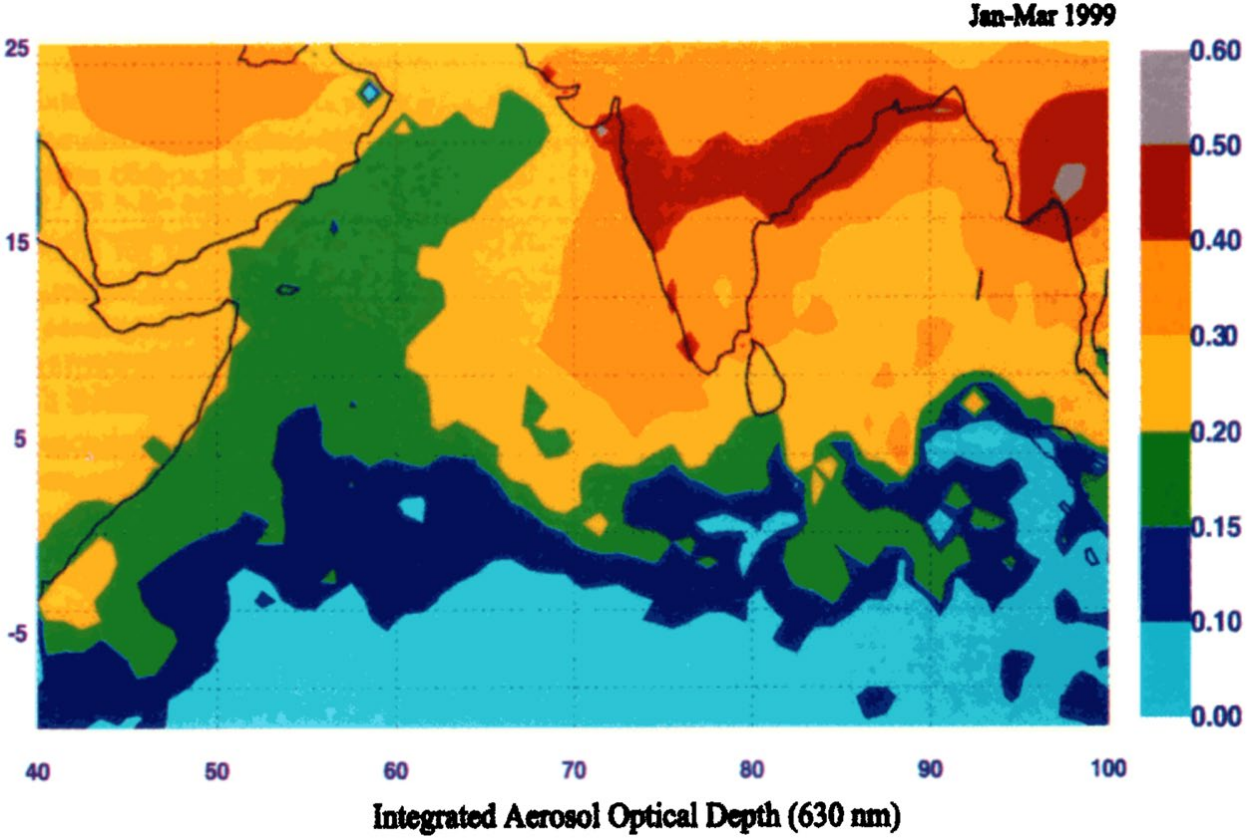
Regional map of aerosol optical depth for the period January to March 1999^9^.

Given that the present study has identified significant regions over India where maximum and minimum surface temperatures have shown a cooling trend from the 1950s to the 2000s, it is noteworthy that another significant study^6^ has indicated that the all-India surface air temperature during the drier part of the year, in the period January through May, (corresponding to the two designated periods in the present study of winter and pre-monsoon) has shown a relative cooling by as much as 0.3 C from the 1950s onward when the global effects of greenhouse gases and natural variability are filtered out from the time series temperature data. Thus, the two studies appear to be reporting the same cooling phenomenon. That study utilized century-long global surface temperature observations^7,8^, as well as long-period observations of both maximum and minimum temperatures from a dense network of Indian stations^4^. These are older data sets as compared to the one used in the present study. The previously cited study^6^ did not distinguish temperature patterns in different geographical regions of India as does the present study, but considered India as a whole. The authors showed that cooling was not observed during the wet season, defined as June through December (corresponding partially to the period of the peak of the monsoon in the present study, extending from July through September). They also showed that the observed cooling during the dry season was not a part of a global cooling pattern. Importantly, they attributed the surface cooling to anthropogenic emissions over the Asian region which produces, particularly in the winter and spring months, a large region of brownish haze over most of the North Indian Ocean and South Asia. This haze region is depicted in Fig. 6, which shows a maximum in aerosol optical depth (AOD) distribution across India and the adjacent ocean regions for the period January to March 1999 based on data taken during the Indian Ocean Experiment (INDOEX)^9^. This region of maximum AOD fits exactly with the region of cooling (or reduced warming) as shown in the previously discussed Figs 2–4.

Research has shown that the haze region is composed of aerosols that absorb solar radiation by the so-called direct aerosol effect, reducing surface insolation by about −10 to −30 W/m^2^ (cooling effect), while increasing absorption in the aerosol layer (0 to 3 km) by about 10 to 30 W/m2 (warming effect)^6^. With regard to cloudiness, a 50-year increase has been reported in cloud fraction over the Northern Indian Ocean but the impact on the radiation balance from this increase in cloudiness, through the two so-called aerosol indirect effects^10,11^, has been shown to remain small in comparison to the aerosol direct effect^6,12^.

**Figure 7:**
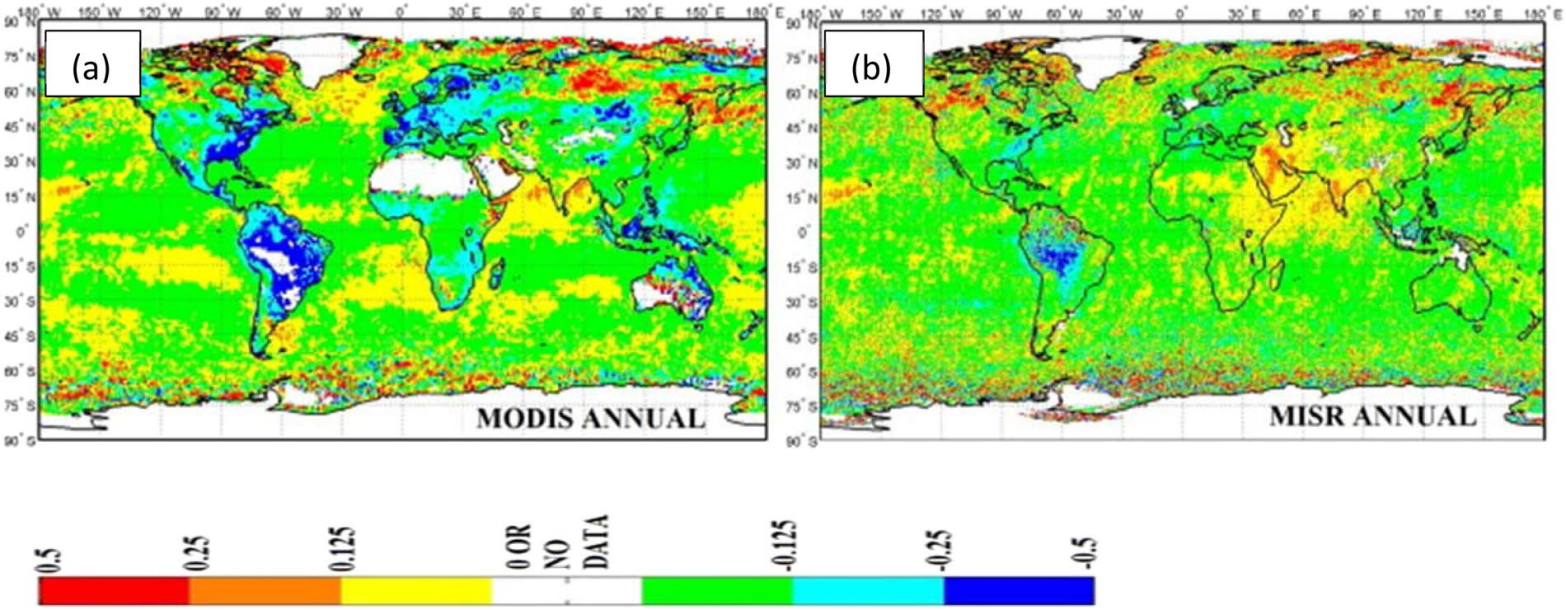
Annual global relative changes in AOD (%). Depictions are from (**a**) MODIS (1 by 1 degree) data and (**b**) MISR (0.5 by 0.5 degree) data. Percent change is based on average AOD for the period 2008–14 minus average AOD for the period 2001–07^15^.

A number of studies have supported the previous findings^6,9^ of the presence of a large haze region over parts of India, composed of aerosols. Satellite observations from MODIS and MISR for the period 2001–2013 showed increasing trends in AOD during parts of the year, due to an increase in anthropogenic activities^13^. Likewise, positive trends in AOD have been reported over the Indian subcontinent and adjacent ocean areas during the period 2003–2014 from MODIS data^14^. At the same time, no significant trends in cloud properties were found except in a few isolated pockets. In a global perspective, MODIS and MISR data for the period of 2001–2014 have shown increasing trends in AOD in the economically growing portions of the Asian landmass and surrounding oceanic regions^15^. This was found to be especially true for the Indian subcontinent and the adjacent Arabian Sea and Bay of Bengal. Figure 7 shows a band of increasing AOD in the annual depiction stretching from northeast India southwestward across the country in precisely the region where cooling (or reduced warming) is found in the previously discussed Figs 2–4, just as with the AOD depiction in Fig. 6. This pattern is most evident in the MISR depiction.

## Hypothesis for the Warming and Cooling Patterns Observed over India

It is very important to consider what might happen to the extra heat that is generated in the aerosol haze layer. It has been suggested that “the climate system could respond by transporting the (heat from) absorbed radiation to regions outside the haze layer and thus warm the surface there. In other words, the region that contributes to the haze will be subject to surface cooling whereas the regions outside could be warming, thus contributing to global warming”^6^.

A Community Climate Model (CCM)^16^ has been used to study the effects of the aerosol-heated layer (700 hPa and below) on the atmospheric circulation in the Indian region, in order to further explore this issue^9^. The model verified that surface cooling occurred below the layer that was heated by aerosol absorption of infrared radiation. In the heated layer, rising motion occurred and moist convection developed as a dynamical response. Compensating sinking occurred to the north and south of the region of rising air, with warming of the surface air due to the adiabatic descent. These, and many other aspects of the interaction of aerosols with the monsoon climate over Asia, are discussed in a recent comprehensive review paper^17^.

**Figure 8:**
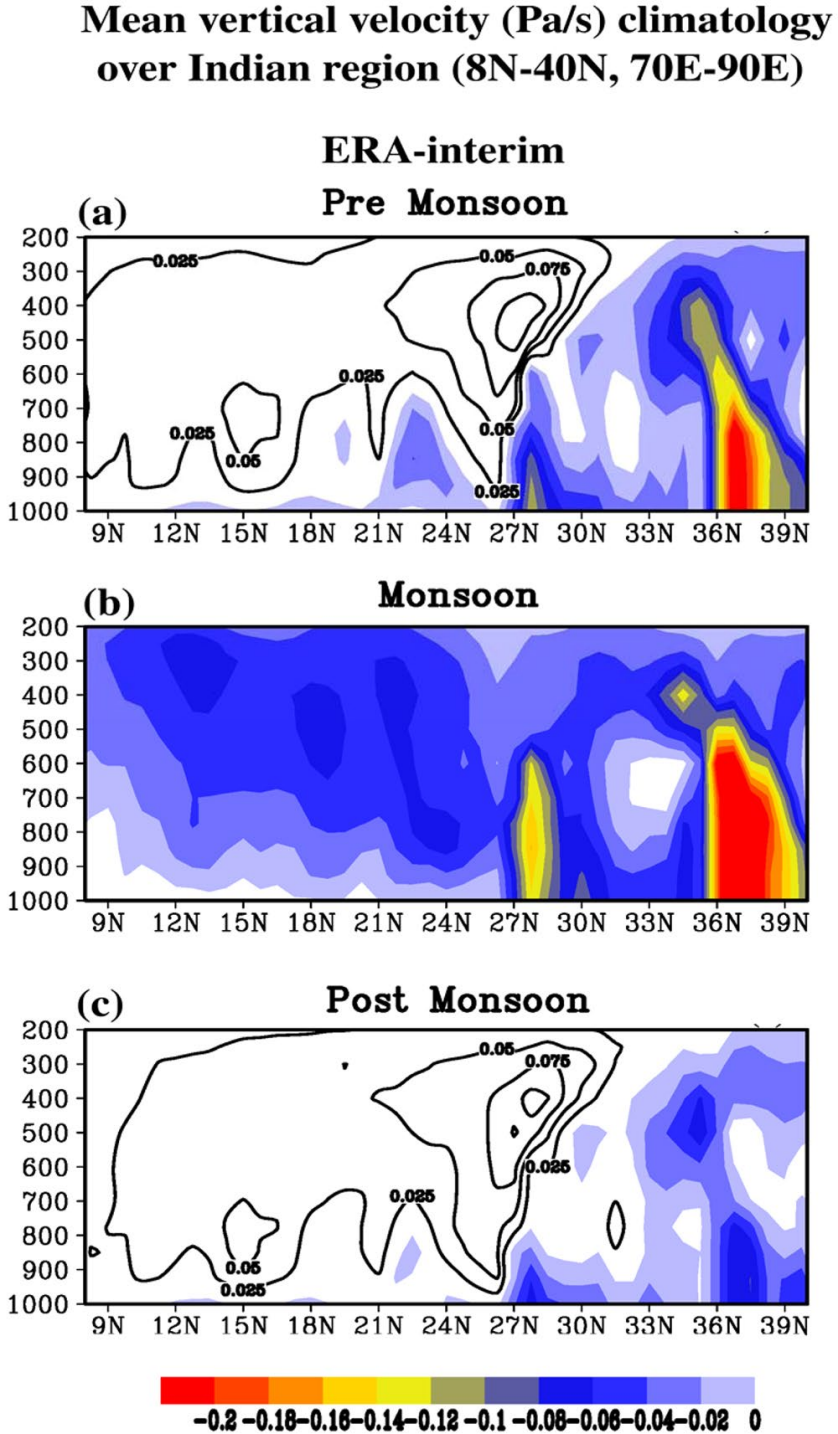
Mean vertical velocity climatology over the Indian region (8N-40N, 70E-90E) from ERA-interim data for the period 1979–2006. Units are Pascals (Pa) per second (s); negative values (upward motion) are shaded while positive values (downward motion) are unshaded. (**a**)Pre-monsoon, (**b**)monsoon, and (**c**)post-monsoon periods are for April 1 – March 31, July 1 – September 30, and January 1 – March 31, respectively, as defined throughout the paper.

To further explore the vertical motion field associated with the aerosol-heated layer, ERA-Interim data for the period 1979–2006 was used in the present study to construct north-south vertical sections of vertical velocity, extending from 8N–40N, averaged over the longitudes 70E–90E, for the pre-monsoon, monsoon, and post-monsoon periods as defined in this paper. This is shown in Fig. 8. In this figure the vertical motion patterns for the pre-monsoon and post-monsoon periods are remarkably similar and they agree with the patterns from the CCM model discussed above. Rising motion is seen around 800 hPa and 23N (blue shading) in both diagrams and this is occurring precisely in the layer where heating is occurring due to absorption of infrared radiation by the aerosols. To the north and south of this region in both diagrams sinking motion is occurring at two latitudes, 26N and 15N, and this agrees with the two regions of compensating sinking motion and surface warming due to descent found in the CCM results. Very significantly, the region of rising motion in the pre-monsoon and post-monsoon diagrams, at about 23N, coincides with the region of surface cooling seen in Figs 2(a–c) and 4(a–c) across central India, and the regions of sinking motion at 26N and 15N coincide with the regions of surface warming found in Figs 2(a–c) and 4(a–c) over northwestern India and southern India, respectively. In Fig. 8b the vertical motion field for the monsoon period shows mostly rising motion throughout, and the previously discussed regions of rising and sinking motion are not found. This is thought to be the case because the extreme uplift of air and heavy rains of the peak of the monsoon overwhelm the vertical motion field associated with the aerosol layer.

The regions of cooling and warming over India which the current study has documented in Figs 2–4 are consistent with the previous findings involving the use of the CCM model^6,9^ and the ERA-interim data used in the current study. Therefore, the concept of the cooling of the surface air beneath the layer of absorbing aerosols, the development of rising warm air above the surface in that region, and the occurrence of adiabatic warming to the north and south of that region due to compensating sinking motion is adopted in the current study as a reasonable hypothesis for the multi-decadal temperature patterns observed over India in Figs 2–4.

## Summary and Discussion

Based on the most comprehensive surface temperature data set for India to date, this study has shown that there has been a multi-decadal increase in maximum surface temperatures over India observed during the pre-monsoon months of April and May when each decade from the 1950s through the 2010s is compared. These results are consistent with IPCC reports detailing a warming of the global climate system which has accelerated since the 1950s. Decade mean maximum surface temperatures over India in the pre-monsoon months in the 1950s showed limited areas with values as high as 40 C (104 F), but by the 2010s the area with values exceeding 40 C had expanded to include the majority of the Indian peninsula, with peak values in south-central India reaching 42 C (108 F). Such extreme values are particularly impressive since they are decadal mean values, implying much higher temperatures on individual days. These findings are highly significant since they are based on a recent comprehensive surface temperature data set incorporating 395 Indian stations, interpolated to a 1-degree latitude by 1-degree longitude grid^3^.

The multi-decadal patterns of surface temperature change over India were explored further in this study by constructing maps of decade mean surface temperatures for the decade of the 2000s minus the decade of the 1950s. Such maps were constructed for maximum, minimum and mean temperature for three periods during the annual monsoon cycle: pre-monsoon period or “summer” (April 1 to May 31), peak of the monsoon period (July 1 to September 30), and “winter,” or the period well past the time of the monsoon (January 1 to March 31). Although subtle differences were noted in comparing maximum, minimum, and mean temperatures within each of these periods, and in comparing maximum, minimum, and mean temperatures between these periods, there was a consistent pattern of warming from the 1950s to the 2000s over northwestern and southern India, and a consistent pattern of cooling (or reduced warming) from the 1950s to the 2000s in a broad zone anchored over northeastern India and extending southwestward across central India. Nearly identical temperature trend patterns were noted for comparisons of the decades of the 2010s to the 1950s, the 2000s to the 1960s, and the 1990s to the 1950s (not shown). These consistent temperature trend patterns were further confirmed by the spatial distribution of annual mean temperature trends for the period 1901–2016, as published by the Indian Meteorological Department.

The present study compares favorably to other studies of long term changes in Indian surface temperatures: diurnal symmetry in maximum and minimum temperatures agrees with other studies^5^; a region of cooling extending from northeastern India southwestward across the country is in broad agreement with an all-India cooling trend observed since the 1950s in the drier part of the year extending from January through May^6^ (corresponding to the periods of “pre-monsoon” and “winter” in the present study).

The hypothesis adopted in this study for explaining the warming and cooling patterns in the decadal -mean temperature patterns over India is based on the presence of a large region of brownish haze that is found over most of the North Indian Ocean and South Asia, particularly in the winter and spring months^6,9,13–15^. The haze region is composed of aerosols, measured in satellite observations as aerosol optical depth (AOD), that absorb solar radiation by the direct aerosol effect, which reduces insolation at the earth’s surface leading to cooling, while increasing absorption in the aerosol layer leading to warming. The region of maximum AOD in Figs 6 and 7 coincides with the region of surface cooling (or reduced warming) depicted in Figs 2–4, and its presence is adopted as a reasonable explanation for the region of cooling (or reduced warming) over India.

The effects of the aerosol-heated layer (700 hPa and below) on the atmospheric circulation in the Indian region was studied using a Community Climate Model (CCM)^9^. The model verified that surface cooling occurred below the layer that was heated by aerosol absorption of infrared radiation. In the heated layer, rising motion and moist convection developed as a dynamical response, with compensating sinking motion occurring to the north and south of the region of rising air leading to warming of the surface air due to the adiabatic descent. This warming from compensating sinking motion is adopted as a reasonable explanation for the two regions of warming found in the present study, one in northwestern India and one in southern India. The vertical motion field described in the CCM study was confirmed in the present study by using ERA-interim data for the period 1979–2006. North-south vertical sections of vertical velocity over India were constructed for pre-monsoon, monsoon, and post-monsoon periods. The vertical motion patterns for the pre-monsoon and post-monsoon periods were found to be remarkably similar and they showed rising motion in the aerosol-heated layer and sinking motion to the north and south of this region, just as in the CCM study. The ERA-interim vertical motion fields for the peak of the monsoon period did not confirm these patterns, as rising motion was seen throughout most of the vertical section. It was suggested that the very strong upward motion associated with the period of heavy monsoon rains was masking the subtle vertical motion field associated with the aerosol layer as seen in the pre-monsoon and post-monsoon periods.

In summary, the regions of cooling and warming over India which the current study has documented are consistent with other studies^6,9^. The concept of the cooling of the surface air beneath the layer of absorbing aerosols, the development of rising air above the surface in that region, and the occurrence of adiabatic warming to the north and south of that region due to compensating sinking motion is adopted in the current study as the most reasonable hypothesis for the multi-decadal temperature patterns observed in Figs 2–4.

**Figure 9:**
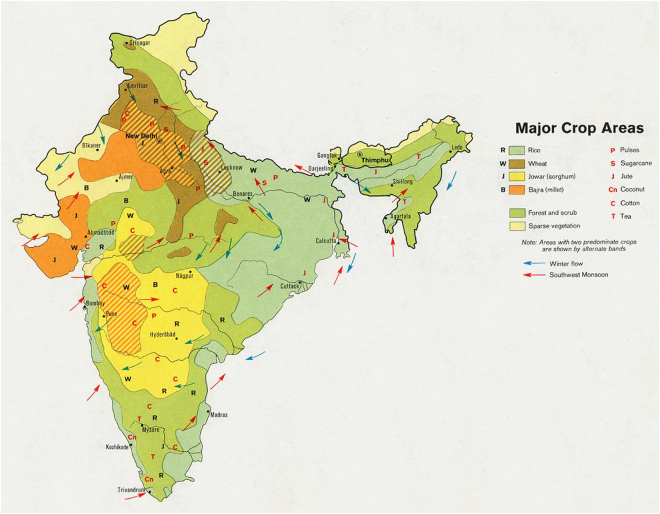
The major crop growing areas of India. Growing regions for the primary crop of rice (light green) and sorghum (yellow) lie in the northeast to southwest oriented zone of cooling (or reduced warming) depicted in Figs. 2–4. (University of Texas Libraries, www.lib.utexas.edu/maps/middle_east_and_asia).

The multi-decadal patterns of temperature change which this study has documented may prove to be very important in planning for the effects of climate change in India, particularly in assessing the impacts of climate change on the agricultural system of the country. As pointed out in the introduction, India is very vulnerable to climate change because its agricultural system must feed a huge population utilizing very limited land and water resources in comparison to its population size. Projections of climate change for the period 2020–2039 indicate a possible reduction in crop yield of 4.5–9%. However, there is potentially encouraging news in the maps of multi-decadal temperature change presented in this study. Figure 9, for example, shows that the primary growing regions for the country’s most important crop of rice (and the secondary crop of sorghum) lie directly in the swath of multi-decadal temperature cooling (or reduced warming) as seen in Figs 2–4. This may indicate that these critical growing areas will experience less stress from the soaring temperatures of global warming than the regions of northwest and southern India, which have been shown in this study to be the primary warming areas in the country. This may indicate more optimistic long-term crop yields than current agricultural projections are indicating.

Future studies are needed to assess the potential impacts of climate change on the agricultural production in India, and these studies should include possible long-term trends in precipitation, evapotranspiration, onshore/offshore low-level winds, cloud cover, etc., all of which can affect surface temperature trends, in addition to radiative effects associated with aerosols. In addition, detailed WRF-CHEM model simulations of the circulation patterns over India in the presence of high aerosol counts are needed to further verify the hypothesis adopted in this study for the multi-decadal patterns of temperature change. Such studies were beyond the scope of the present research, whose main focus was on documenting temperature trends from the new high-resolution temperature data set.

## Electronic supplementary material

Figure S1. All India mean temperature anomalies
