# Supplementary material for: Decadal surface temperature trends in India based on a new high-resolution data set
Source: Sci Rep. 2018 May 10;8:7452. doi: 10.1038/s41598-018-25347-2 (PMC5945614; doi:10.1038/s41598-018-25347-2)

Supplementary information

Manuscript title: Decadal surface temperature trends in India based on a new high-resolution data set

Authors: Robert S. Ross, T. N. Krishnamurti, Sandeep Pattnaik, and D. S. Pai

Figure S1. All India mean temperature anomalies. Period is 1901-2016 for **(A)** annual, **(B)** winter, **(C)** pre-monsoon, **(D)** monsoon, and **(E)** post-monsoon. The solid blue curve carries sub-decadal time scale variations smoothed with a binomial filter. Departures are from the 1971-2000 average. India Meteorological Department, Annual Climate Summary 2016, Pune, India.

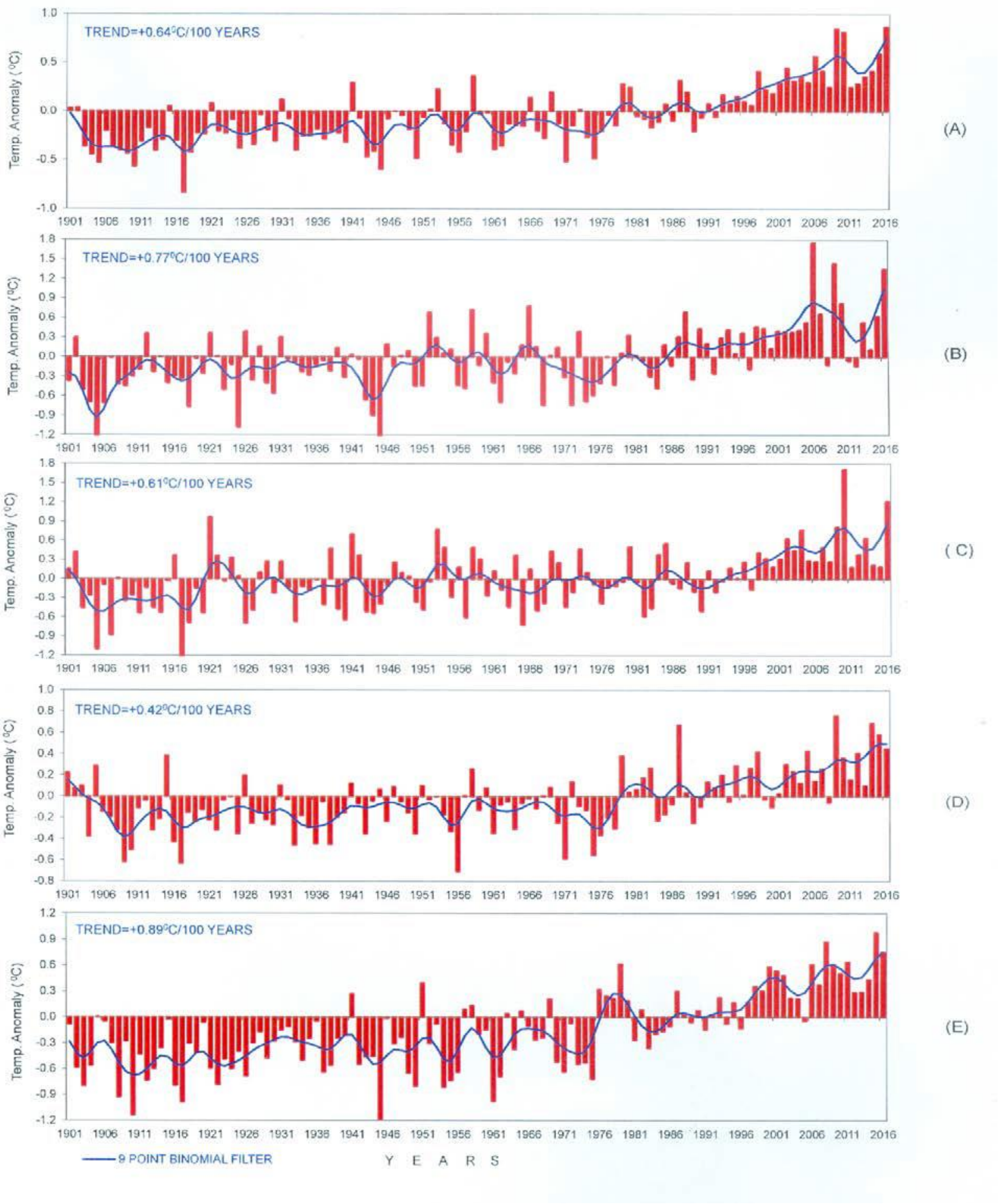

Supplement: Supplementary file 1 — Figure S1. All India mean temperature anomalies [file 41598_2018_25347_MOESM1_ESM.pdf]
